# The Boundary Proportion Differential Control Method of Micro-Deformable Manipulator with Compensator Based on Partial Differential Equation Dynamic Model

**DOI:** 10.3390/mi12070799

**Published:** 2021-07-05

**Authors:** Xiangli Pei, Ying Tian, Minglu Zhang, Ruizhuo Shi

**Affiliations:** School of Mechanical Engineering, Hebei University of Technology, Tianjin 300000, China; pxl_hebut@163.com (X.P.); zhangml@hebut.edu.cn (M.Z.); srzhbgydx@163.com (R.S.)

**Keywords:** micro-deformable manipulator, partial differential equation dynamic model, radial basis function neural network compensator, boundary proportional differential control method

## Abstract

It is challenging to accurately judge the actual end position of the manipulator—regarded as a rigid body—due to the influence of micro-deformation. Its precise and efficient control is a crucial problem. To solve the problem, the Hamilton principle was used to establish the partial differential equation (PDE) dynamic model of the manipulator system based on the infinite dimension of the working environment interference and the manipulator space. Hence, it resolves the common overflow instability problem in the micro-deformable manipulator system modeling. Furthermore, an infinite-dimensional radial basis function neural network compensator suitable for the dynamic model was proposed to compensate for boundary and uncertain external interference. Based on this compensation method, a distributed boundary proportional differential control method was designed to improve control accuracy and speed. The effectiveness of the proposed model and method was verified by theoretical analysis, numerical simulation, and experimental verification. The results show that the proposed method can effectively improve the response speed while ensuring accuracy.

## 1. Introduction

With the rapid development of modern manipulators, the traditional rigid manipulators with large mass and margin cannot satisfy fast response and accurate positioning requirements. However, micro-deformable manipulators have lighter weight, lower energy consumption, and less inertia than traditional manipulators; moreover, they have high precision, high efficiency, high speed, high flexibility, high adaptability, and intelligence [1]. Their application range is wider than that of rigid manipulators. Therefore, the lightweight and dexterous micro-deformable manipulator dynamic model and its precise control have gradually become a hot research topic [2]. 

Researchers have done much work on the dynamic modeling and precise control of micro-deformable manipulators. In [3], to control a single-link flexible manipulator, a hybrid method combining sliding mode and H−∞ theory was proposed. Furthermore, a linear optimal damping controller was used in [4] to adjust the flexible boom vibration mode to a lower level. Based on the partial differential equation (PDE) dynamic model of the flexible manipulator system, some researchers used the adaptive boundary control method [5,6,7] to control the manipulator, while others used the neural network control method [8]. Several researchers have combined the adaptive boundary control method with the approximation or compensation results of radial basis function (RBF) neural network [9,10,11,12,13] to optimize the control performance of flexible systems. Additionally, the RBF neural network proportional differential (PD) control method of the flexible manipulator was studied in [14]. Moreover, in [15], based on a wavelet neural network, a dynamic surface control method was proposed. Furthermore, the iterative learning-based adaptive control methods were designed in [16,17] to obtain trajectory tracking and vibration reduction. The dynamic model of the flexible manipulator system was established, and the dynamic characteristics were analyzed in addition to different methods in [18,19,20]. In [21,22], the boundary control of flexible Timoshenko arm was studied and analyzed. Furthermore, the position control method and residual vibration of the flexible system were analyzed in [23,24,25]. Moreover, some optimized fuzzy control methods were proposed in [26,27,28]. However, most studies mentioned above simplified the micro-deformation manipulator into a relatively simple ideal model; these idealized models were not consistent with the actual situation [6,8,17,23]. Additionally, most studies ignored the infinite dimensionality of the micro-deformation manipulator. These lead to overflow and instability; moreover, change of the system space state cannot be described accurately [18,19,26]. The overflow problems were considered in a few PDE modeling papers [5,7,9,12] and RBF neural network was used to estimate interference of one end, but the other end was ignored [9]. Due to the partial interference being neglected, the established model was not accurate enough. This study completely considered the interference at both ends of the boundary while establishing the PDE dynamic model. The estimated interference results were added to the control law using two outputs RBF neural network for improvement and optimization purposes. Thus, the accuracy of the model was improved, and the model fitted the actual situation better.

Furthermore, this study fully considered the vibration and deformation of the micro-deformation manipulator and the joint micro-deformation. Moreover, we studied the distributed parameter boundary PD control method of RBF neural network compensator for the micro-deformation manipulator. First, the infinite-dimensional PDE dynamic model of the micro-deformable manipulator system was established. After that, the PD control method and stability analysis of the micro-deformable manipulator based on the RBF neural network compensator were introduced. Finally, numerical simulation and analysis of the control method were performed. Additionally, the response speed of the proposed method was increased by at least 30% compared with the adaptive boundary control method and RBF neural network method. Experimental verification was carried out for the proposed method at last.

## 2. Dynamic Modeling of Micro-Deformable Manipulator

An accurate dynamic model is a basis for achieving high-performance control. The control methods of micro-deformation manipulators were developed based on the ordinary differential equation (ODE) dynamic model in most studies. However, it cannot accurately describe the distributed parameter characteristics of the micro-deformable structure and may cause overflow instability problems. The PDE dynamic model can reflect the dynamic characteristics of the micro-deformed structure more accurately than the ODE dynamic model [5,9]. The Hamilton method was used to derive the PDE dynamic equation of the micro-deformable manipulator system [5,7,9,12]; moreover, the corresponding boundary conditions of the system were obtained. This process did not require complex force analysis of the micro-deformable manipulator system. The dynamic model can be derived directly by mathematical methods.

**Remark** **1.** *The deformation of the micro-deformable manipulator is spatiotemporal, but the time variable t does not affect the calculation and derivation of the dynamic model. For simplicity, we omitted the time variable, t, in the function variable. For example,* y(x,t)* is expressed as* y(x)*,* l(x,t)* is expressed as* l(x)*, and* θ(t)* is expressed as* θ.

**Remark** **2.**  *The usage and explanation of subscripts in this paper are as follows:*

${\left(*\right)}_{x}=\frac{\partial \left(*\right)}{\partial x}$, ${\left(*\right)}_{xx}=\frac{\partial^{2}\left(*\right)}{\partial x^{2}}$, ${\left(*\right)}_{xxx}=\frac{\partial^{3}\left(*\right)}{\partial x^{3}}$, ${\left(*\right)}_{xxxx}=\frac{\partial^{4}\left(*\right)}{\partial x^{4}}$, ${\left(*\right)}_{t}=\frac{\partial \left(*\right)}{\partial t}$, ${\left(*\right)}_{tt}=\frac{\partial^{2}\left(*\right)}{\partial t^{2}}$, ${\left(*\right)}_{xtt}=\frac{\partial \left(*\right)}{\partial x\partial t^{2}}$.

**Figure 1:**
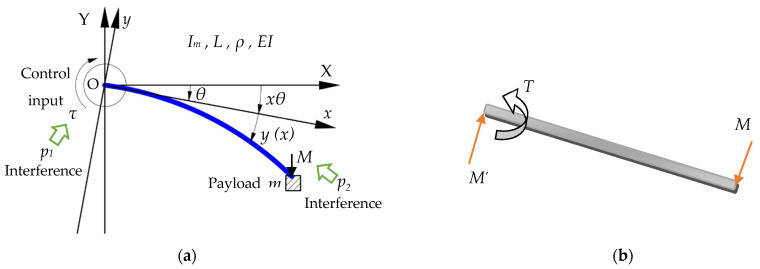
A typical single link micro-deformable manipulator: (**a**) coordinate diagram; (**b**) free-body diagram of the system.

We considered the plane motion single-rod micro-deformable manipulator as the research object (Figure 1); its cross-section was circular, and its radius, R, was 0.01 *m*. The relevant symbols of the manipulator are shown in Nomenclature. According to the theory and formulas of structural dynamics, mean normal stress ratio to shear stress can be calculated, as shown in Equation (1). Therefore, $\sigma_{ave}\gg \tau_{s}$. In other words, bending deformation is the main deformation; therefore, the shear deformation can be neglected. Hence, we developed the dynamic model based on the Euler–Bernoulli beam theory.

(1) $$\frac{\sigma_{ave}}{\tau_{s}}=\frac{\sigma_{\max}\cdot \left(2/3\right)}{\tau_{s}}=\frac{\left(MLy_{\max}/I_{z}\right)\cdot \left(2/3\right)}{M/A}=\frac{8M/3\pi R^{3}}{M/\pi R^{2}}=\frac{8}{3R}\approx 266.67$$

The offset of any point, [x,y(x)], in the follow-up coordinate system, xOy, from the micro-deformation manipulator in the inertial coordinate system, XOY, is approximately expressed as l(x).

(2) l(x)=xθ+y(x)

The manipulator is regarded as an Euler–Bernoulli beam here and clamped to a motor at x. The natural boundary conditions can be expressed as:

(3) $$y\left(0\right)=y_{x}\left(0\right)=0$$

Using Equations (2) and (3), we get:

(4) $$l\left(0\right)=0,l_{x}\left(0\right)=\theta ,\frac{\partial^{m}l\left(x\right)}{\partial x^{m}}=\frac{\partial^{m}y\left(x\right)}{\partial x^{m}},m\geq 2$$

According to Hamilton principle [5,6,29,30], the PDE dynamic equations of the micro-deformable manipulator can be developed for every $0\leq t_{1}<t<t_{2}$, as shown in Equation (5):

(5) $$\int_{t_{1}}^{t_{2}}\left(\delta W_{k}-\delta W_{p}+\delta W_{nc}\right)dt=0$$

where $\delta W_{k}$, $\delta W_{p}$ and $\delta W_{nc}$ represent the kinetic energy, potential energy, and the variation of work done by non-conservative force, respectively; $t_{i}$ denotes a moment.

The total kinetic energy of the system can be obtained considering the rotational kinetic energy of the micro-deformation manipulator joint, the kinetic energy of the manipulator, and the kinetic energy of the load, as shown in Equation (6).

(6) $$W_{k}=\frac{1}{2}I_{m}\theta_{t}^{2}+\frac{1}{2}\int_{0}^{L}\rho l_{t}^{2}\left(x\right)dx+\frac{1}{2}ml_{t}^{2}\left(L\right)$$

The potential energy of the system is expressed as:

(7) $$W_{p}=\frac{1}{2}\int_{0}^{L}EIy_{xx}^{2}\left(x\right)dx$$

The work done by the non-conservative force of the system is expressed as:

(8) $$W_{nc}=\tau \theta +p_{1}\theta +Ml\left(L\right)+p_{2}l\left(L\right)$$

Substituting Equations (4), (6)–(8) into Equation (5), we get:

(9) $$\begin{array}{l} \int_{t_{1}}^{t_{2}}\left(\delta W_{k}-\delta W_{p}+\delta W_{nc}\right)\\ =-\int_{t_{1}}^{t_{2}}\int_{0}^{L}K_{1}\delta l\left(x\right)dxdt-\int_{t_{1}}^{t_{2}}K_{2}\delta l_{x}\left(0\right)dt-\int_{t_{1}}^{t_{2}}K_{3}\delta l\left(L\right)dt-\int_{t_{1}}^{t_{2}}K_{4}\delta l_{x}\left(L\right)dt=0 \end{array}$$

where:

$$\left\{ \begin{array}{l} K_{1}=\rho l_{tt}\left(x\right)+EIl_{xxxx}\left(x\right)\\ K_{2}=I_{m}l_{xtt}\left(0\right)-EIl_{xx}\left(0\right)-\left(\tau +p_{1}\right)\\ K_{3}=ml_{tt}\left(L\right)-EIl_{xxx}\left(L\right)-\left(M+p_{2}\right)\\ K_{4}=EIl_{xx}\left(L\right) \end{array}\right.$$

Each monomial in Equation (9) is linearly independent because δl(x), $\delta l_{x}\left(0\right)$, δl(L), $\delta l_{x}\left(L\right)$ are independent variables. Hence, $K_{1}=K_{2}=K_{3}=K_{4}=0$. Therefore, the PDE dynamic model of the micro-deformation manipulator system is obtained as Equation (10).

(10) $$\left\{ \begin{array}{l} \rho l_{tt}\left(x\right)=-EIl_{xxxx}\left(x\right)\\ \tau =I_{m}l_{xtt}\left(0\right)-EIl_{xx}\left(0\right)-p_{1}\\ M=ml_{tt}\left(L\right)-EIl_{xxx}\left(L\right)-p_{2}\\ l_{xx}\left(L\right)=0 \end{array}\right.$$

where: $l_{tt}\left(x\right)=x\theta_{tt}+y_{tt}\left(x\right)$.

The dynamic model of the manipulator system was developed from the mathematical model perspective. The motion characteristics are related to time and position; therefore, the micro-deformable manipulator is essentially a distributed parameter system. Hence, the distributed parameter model was established based on the PDE equation. Furthermore, the corresponding control method adopted the distributed parameter boundary PD control, which can effectively obtain the micro-deformable system control. The boundary control only needs a small number of thrusters to achieve a better control effect than the discrete distributed control.

## 3. RBF Neural Network Distributed Boundary PD Control Method

**Figure 2:**
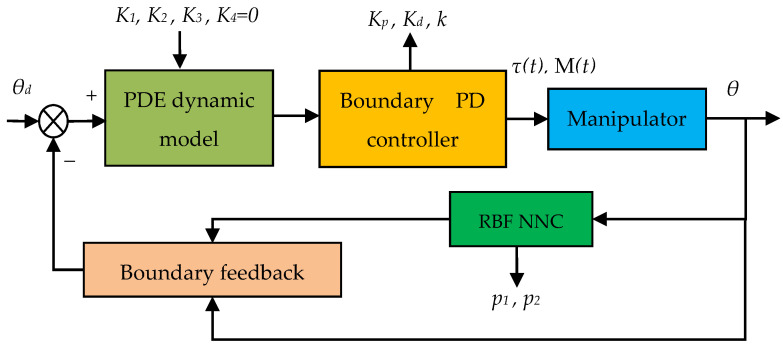
Boundary PD control principle with RBF neural network compensator.

The paper [5] proposed an adaptive boundary control method suitable for the PDE dynamic model. The method was simple and accurate, but the response time remained long. Compared with other machine learning algorithms, RBF neural network has the ability of parallel information processing, stronger computing power, and faster running speed. Moreover, RBF neural network can avoid the local minimum problem. Therefore, the control scheme based on RBF neural network is more suitable for the requirement of real-time control. Thus, RBF neural network compensator was used to improve the adaptive boundary control method described in [5]. The joint position was adjusted, and the vibration was weakened by designing the Lyapunov function and boundary PD control law (Figure 2).

### 3.1. Design of Distributed Boundary PD Control Law Based on RBF Neural Network Compensator

The error function is defined as $e=\theta -\theta_{d}$, where $\theta_{d}$ denotes the expectation angle. Consider that the uncertainty interference $p_{1}$ and $p_{2}$ of the actual model is unknown. The RBF neural network is used to estimate the uncertainty interference $p_{1}$ and $p_{2}$. The estimated values are ${\hat{p}}_{1}$ and ${\hat{p}}_{2}$. Furthermore, the input vector of the neural network is considered as $x=\left[\begin{array}{ccccc} e^{T} & {\dot{e}}^{T} & \theta_{d}^{T} & {\dot{\theta }}_{d}^{T} & {\ddot{\theta }}_{d}^{T} \end{array}\right]$. Moreover, the ideal RBF neural network algorithm is shown in Equation (11).

(11) $$\left\{ \begin{array}{l} \varphi_{i}=\exp\left(-\frac{{\left\|x-c_{i}\right\|}^{2}}{2b_{i}^{2}}\right),i=1,2,\ldots ,m\\ p_{1}=W_{1}^{*}\varphi_{1}\left(x\right)+\gamma_{1},p_{2}=W_{2}^{*}\varphi_{2}\left(x\right)+\gamma_{2} \end{array}\right.$$

where $\varphi_{i}={\left[\varphi_{1},\varphi_{2},\ldots ,\varphi_{m}\right]}^{T}$ denotes the output vector of hidden layer obtained by Gaussian function, *m* represents the number of neurons in the hidden layer, $c_{i}$ is the coordinate vector value of the Gaussian function’s center point, $b_{i}$ represents the width of Gaussian function, $W_{i}^{*}$ denotes the ideal weight matrix of the neural network, and $\gamma_{i}$ is the network estimation error.

The output of the RBF neural network is shown in Equation (12).

(12) $$\left\{ \begin{array}{l} {\hat{p}}_{1}={\hat{W}}_{1}^{*}\varphi_{1}\left(x\right)\\ {\hat{p}}_{2}={\hat{W}}_{2}^{*}\varphi_{2}\left(x\right) \end{array}\right.$$

where ${\hat{W}}_{i}^{*}$ represents the estimated weight of the neural network for unknown parameter estimation.

The errors of the above estimation results are defined in Equation (13).

(13) $$\left\{ \begin{array}{l} {\tilde{W}}_{1}^{*}=W_{1}^{*}-{\hat{W}}_{1}^{*},{\tilde{W}}_{2}^{*}=W_{2}^{*}-{\hat{W}}_{2}^{*}\\ {\tilde{p}}_{1}=p_{1}-{\hat{p}}_{1},{\tilde{p}}_{2}=p_{2}-{\hat{p}}_{2} \end{array}\right.$$

Equations (11)–(13) suggest that

(14) $$\left\{ \begin{array}{l} {\tilde{p}}_{1}={\tilde{W}}_{1}^{*}\varphi_{1}\left(x\right)+\gamma_{1}\\ {\tilde{p}}_{2}={\tilde{W}}_{2}^{*}\varphi_{2}\left(x\right)+\gamma_{2} \end{array}\right.$$

Furthermore, the estimated results, ${\hat{p}}_{1}$ and ${\hat{p}}_{2}$, of the RBF neural network were added into the control law as a compensator to compensate for the external interference, in order to obtain the angular response of the micro-deformation manipulator and suppress its deformation and vibration effectively. The RBF-based boundary PD control law is presented as:

(15) $$\left\{ \begin{array}{l} \tau =-k_{p}e-k_{d}e_{t}+{\hat{p}}_{1}\\ M=-ku_{\alpha }+ml_{xxxt}\left(L\right)+{\hat{p}}_{2} \end{array}\right.$$

where: $k_{p}$, $k_{d}$, and k are control gain values and $k_{p}>0$, $k_{d}>0$, k>0, $u_{\alpha }=l_{t}\left(L\right)-l_{xxx}\left(L\right)$, $e_{t}=\theta_{t}$, $e_{tt}=\theta_{tt}$.

**Remark** **3.**  *All parameters mentioned in the designed control law (Equation (15)) are measurable or computable. We can use the position sensor, tachometer, laser displacement sensor, and shear force sensor to get the measurements,* θ, $\theta_{t}$, l(x), and $l_{xxx}\left(x\right)$, respectively. e, $e_{t}$, $e_{tt}$, $l_{t}\left(x\right)$, and $l_{xxxt}\left(x\right)$* can be calculated from the measurements.*

### 3.2. Stability Analysis Based on Lyapunov Function

**Lemma** **1.**  *For* $\phi_{1}\left(x,t\right)$ *,* $\phi_{2}\left(x,t\right)\in R$ *,* x∈[0,L] *,* t∈[0,∞) *, the following inequality holds:*

$$\begin{array}{l} \phi_{1}\left(x,t\right)\phi_{2}\left(x,t\right)\leq \left|\phi_{1}\left(x,t\right)\phi_{2}\left(x,t\right)\right|\leq \phi_{1}{}^{2}\left(x,t\right)+\phi_{2}{}^{2}\left(x,t\right)\\ \left|\phi_{1}\left(x,t\right)\phi_{2}\left(x,t\right)\right|\leq \frac{1}{\lambda }\phi_{1}{}^{2}\left(x,t\right)+\lambda \phi_{2}{}^{2}\left(x,t\right),\left(\lambda >0\right) \end{array}$$

**Lemma** **2.**  *For* p(x,t)∈R*,*x∈[0,L]*,*t∈[0,∞)*, if*p(0,t)=0*,*t∈[0,∞)*, then *$p^{2}\left(x,t\right)\leq L\int_{0}^{L}p_{x}^{2}\left(x,t\right)dx$*,*x∈[0,L]*. Similarly, if*$p_{x}\left(0,t\right)=0$*,*t∈[0,∞)*, then*$p_{x}^{2}\left(x,t\right)\leq L\int_{0}^{L}p_{xx}^{2}\left(x,t\right)dx$*,*x∈[0,L].

**Lemma** **3.**  *For* $V:\left[0,\infty \right)\in R,t\geq t_{0}\geq 0$, if $\dot{V}\leq -\eta V+g$*, then:*

$$V\left(t\right)\leq e^{-\eta \left(t-t_{0}\right)}V\left(t_{0}\right)+\int_{t_{0}}^{t}e^{-\eta \left(t-s\right)}g\left(s\right)ds,\eta >0.$$

**Theorem** **1.** *The closed-loop system described by Equation (10) is asymptotically stable, with the proposed RBF neural network compensator (Equation (12)) and control law (Equation (15)), namely,* $\theta \to \theta_{d}$, $\theta_{t}\to 0$, y(x)→0, $y_{t}\left(x\right)\to 0$, as x∈[0,L], t→∞.

**Proof** **of** **Theorem** **1.** *The Lyapunov function is defined to prove the stability of the controller, as in Equation (16).
*

(16) $$H\left(t\right)=W_{1}+W_{2}+W_{\alpha }$$

□ 

where:

(17) $$\left\{ \begin{array}{l} W_{1}=\frac{1}{2}\int_{0}^{L}\rho l_{t}^{2}\left(x\right)dx+\frac{1}{2}EI\int_{0}^{L}y_{xx}^{2}\left(x\right)\\ W_{2}=\frac{1}{2}I_{m}e_{t}^{2}+\frac{1}{2}k_{p}e^{2}+\frac{1}{2}mu_{\alpha }^{2}-p_{1}e-p_{2}\int_{t_{1}}^{t_{2}}u_{\alpha }dt\\ W_{\alpha }=\alpha \rho \int_{0}^{L}xl_{t}\left(x\right)le_{x}\left(x\right)dx+\alpha I_{m}ee_{t} \end{array}\right.$$

In Equation (17), $W_{1}$ represents the inhibition index bending deformation of the micro-deformation manipulator. The first two items in $W_{2}$ represent the control error-index, and the rest of the items are the auxiliary terms. Moreover, $W_{\alpha }$ is the auxiliary part and α denotes a small positive constant. Furthermore,

(18) $$le\left(x\right)=xe+y\left(x\right),\;le_{x}\left(x\right)=e+y_{x}\left(x\right),\;le_{xx}\left(x\right)=y_{xx}\left(x\right)=l_{xx}\left(x\right)$$

According to Lemmas 1 and 2, we obtain:

(19) $$\begin{array}{l} \left|W_{\alpha }\right|\leq \alpha \rho L\int_{0}^{L}l_{t}{}^{2}\left(x\right)dx+2\alpha \rho L^{2}e^{2}+2\alpha \rho L^{3}\int_{0}^{L}l_{xx}^{2}\left(x\right)dx+\alpha I_{m}\left(e^{2}+e_{t}^{2}\right)\\ \leq \alpha_{1}\left(W_{1}+W_{2}\right) \end{array}$$

where: $\alpha_{1}=\max\left(2\alpha L,2\alpha \rho L^{3}/EI,2\left(\alpha I_{m}+2\alpha \rho L^{2}\right)/k_{p},2\alpha \right)$.

Therefore,

(20) $$-\alpha_{1}\left(W_{1}+W_{2}\right)\leq W_{\alpha }\leq \alpha_{1}\left(W_{1}+W_{2}\right)$$

Take 0 < *α*_1_ < 1, i.e., $0<\max\left(2\alpha L,2\alpha \rho L^{3}/EI,2\left(\alpha I_{m}+2\alpha \rho L^{2}\right)/k_{p},2\alpha \right)<1$, then α can be designed as $0<\alpha <\frac{1}{\left(2L,2\rho L^{3}/EI,2\left(I_{m}+2\rho L^{2}\right)/k_{p},2\right)}$. We define $1>\alpha_{2}=1-\alpha_{1}>0$, $2>\alpha_{3}=1+\alpha_{1}>1$; hence,

(21) $$0\leq \alpha_{2}\left(W_{1}+W_{2}\right)\leq H\left(t\right)\leq \alpha_{3}\left(W_{1}+W_{2}\right)$$

According to inequality in Equation (21), the Lyapunov function is positively definite. Hence,

(22) $$H_{t}\left(t\right)={\left(W_{1}\right)}_{t}+{\left(W_{2}\right)}_{t}+{\left(W_{\alpha }\right)}_{t}$$

In Equation (22),

(23) $$\left\{ \begin{array}{l} {\left(W_{1}\right)}_{t}=\int_{0}^{L}\rho l_{t}\left(x\right)l_{tt}\left(x\right)dx+EI\int_{0}^{L}y_{xx}\left(x\right)y_{xxt}\left(x\right)\\ {\left(W_{2}\right)}_{t}=I_{m}e_{t}e_{tt}+k_{p}ee_{t}+mu_{\alpha }{\left(u_{\alpha }\right)}_{t}-p_{1}e_{t}-p_{2}u_{\alpha }\\ {\left(W_{\alpha }\right)}_{t}={\left(W_{\alpha 1}\right)}_{t}+{\left(W_{\alpha 2}\right)}_{t}+{\left(W_{\alpha 3}\right)}_{t} \end{array}\right.$$

In Equation (23),

(24) $$\left\{ \begin{array}{l} {\left(W_{\alpha 1}\right)}_{t}=\alpha \rho \int_{0}^{L}xl_{tt}\left(x\right)le_{x}\left(x\right)dx\\ {\left(W_{\alpha 2}\right)}_{t}=\alpha \rho \int_{0}^{L}xl_{t}\left(x\right)l_{t}e_{x}\left(x\right)dx\\ {\left(W_{\alpha 3}\right)}_{t}=\alpha I_{m}\left(e_{t}^{2}+ee_{tt}\right) \end{array}\right.$$

Substituting Equation (10) into Equation (23) and combining Equation (4), ${\left(W_{1}\right)}_{t}$ can be rewritten as:

(25) $${\left(W_{1}\right)}_{t}=-EIy_{xxx}\left(L\right)l_{t}\left(L\right)-EIy_{xx}\left(0\right)\theta_{t}=-EIl_{xx}\left(0\right)e_{t}-EIl_{xxx}^{2}\left(L\right)-EIl_{xxx}\left(L\right)u_{\alpha }$$

Using Equations (10) and (15) and combining Equations (23) and (25), we obtain:

(26) $$\begin{array}{l} {\left(W_{1}\right)}_{t}+{\left(W_{2}\right)}_{t}=-EIl_{xx}\left(0\right)e_{t}-EIl_{xxx}^{2}\left(L\right)-EIl_{xxx}\left(L\right)u_{\alpha }+I_{m}e_{t}e_{tt}+k_{p}ee_{t}+mu_{\alpha }{\left(u_{\alpha }\right)}_{t}-p_{1}e_{t}-p_{2}u_{\alpha }\\ =e_{t}\left(-EIl_{xx}\left(0\right)+I_{m}e_{tt}+k_{p}e-p_{1}\right)+u_{\alpha }\left(-EIl_{xxx}\left(L\right)+m{\left(u_{\alpha }\right)}_{t}-p_{2}\right)-EIl_{xxx}^{2}\left(L\right)\\ =e_{t}\left(\tau +k_{p}e-p_{1}\right)+u_{\alpha }\left(M-ml_{xxxt}\left(L\right)-p_{2}\right)-EIl_{xxx}^{2}\left(L\right)\\ =-k_{d}e_{t}^{2}-ku_{\alpha }^{2}-EIl_{xxx}^{2}\left(L\right) \end{array}$$

According to Lemmas 1–3, combining Equations (10) and (18), and using the method of integral by parts, we deduce:

(27) $$\begin{array}{l} {\left(W_{\alpha 1}\right)}_{t}=-\alpha EILle_{x}\left(L\right)l_{xxx}\left(L\right)-\frac{3}{2}\alpha EI\int_{0}^{L}l_{xx}^{2}\left(x\right)dx-\alpha EIel_{xx}\left(0\right)\\ \leq -\left(\frac{3}{2}\alpha -2\alpha L^{2}-\frac{2\alpha L^{3}}{EI}\right)\int_{0}^{L}EIl_{xx}^{2}\left(x\right)dx+\alpha EILl_{xxx}^{2}\left(L\right)-\alpha EIel_{xx}\left(0\right)+\left(2\alpha EIL+2\alpha L^{2}\right)e^{2} \end{array}$$

(28) $${\left(W_{\alpha 2}\right)}_{t}=\frac{1}{2}\alpha \rho Ll_{t}^{2}\left(L\right)-\frac{1}{2}\alpha \rho \int_{0}^{L}l_{t}^{2}\left(x\right)dx$$

(29) $${\left(W_{\alpha 3}\right)}_{t}=\alpha I_{m}e_{t}^{2}+\alpha eEIl_{xx}\left(0\right)-\alpha k_{p}e^{2}-k_{d}\alpha ee_{t}\leq \left(\alpha I_{m}+k_{d}\alpha \right)e_{t}^{2}-\left(\alpha k_{p}-k_{d}\alpha \right)e^{2}+\alpha eEIl_{xx}\left(0\right)$$

We obtain Equation (30) using Equations (23), (27)–(29).

(30) $$\begin{array}{l} {\left(W_{\alpha }\right)}_{t}\leq -\left(\frac{3}{2}\alpha -2\alpha L^{2}-\frac{2\alpha L^{3}}{EI}\right)\int_{0}^{L}EIl_{xx}^{2}\left(x\right)dx+\alpha EILl_{xxx}^{2}\left(L\right)+\left(2\alpha EIL+2\alpha L^{2}\right)e^{2}\\ +\frac{1}{2}\alpha \rho Ll_{t}^{2}\left(L\right)-\frac{1}{2}\alpha \rho \int_{0}^{L}l_{t}^{2}\left(x\right)dx+\left(\alpha I_{m}+k_{d}\alpha \right)e_{t}^{2}-\left(\alpha k_{p}-k_{d}\alpha \right)e^{2} \end{array}$$

Substituting Equations (26) and (30) into Equation (22), we get Equation (31).

(31) $$\begin{array}{l} H_{t}\left(t\right)={\left(W_{1}\right)}_{t}+{\left(W_{2}\right)}_{t}+{\left(W_{\alpha }\right)}_{t}\\ \leq -k_{d}e_{t}^{2}-ku_{\alpha }^{2}-EIl_{xxx}^{2}\left(L\right)-\left(\frac{3}{2}\alpha -2\alpha L^{2}-\frac{2\alpha L^{3}}{EI}\right)\int_{0}^{L}EIl_{xx}^{2}\left(x\right)dx+\alpha EILl_{xxx}^{2}\left(L\right)\\ +\left(2\alpha EIL+2\alpha L^{2}\right)e^{2}+\frac{1}{2}\alpha \rho Ll_{t}^{2}\left(L\right)-\frac{1}{2}\alpha \rho \int_{0}^{L}l_{t}^{2}\left(x\right)dx+\left(\alpha I_{m}+k_{d}\alpha \right)e_{t}^{2}-\left(\alpha k_{p}-k_{d}\alpha \right)e^{2}\\ =-\left(\frac{3}{2}\alpha -2\alpha L^{2}-\frac{2\alpha L^{3}}{EI}\right)\int_{0}^{L}EIl_{xx}^{2}\left(x\right)dx-\frac{1}{2}\alpha \int_{0}^{L}\rho l_{t}^{2}\left(x\right)dx-\left(k_{d}-\alpha I_{m}-k_{d}\alpha \right)e_{t}^{2}\\ -\left(\alpha k_{p}-k_{d}\alpha -2\alpha EIL-2\alpha L^{2}\right)e^{2}-ku_{\alpha }^{2}+\frac{1}{2}\alpha \rho Ll_{t}^{2}\left(L\right)-\left(EI-\alpha EIL\right)l_{xxx}^{2}\left(L\right) \end{array}$$

The inequality $EI-\alpha EIL>\frac{1}{2}\alpha \rho L$ can be established by choosing *α*, which guarantees that:

(32) $$\frac{1}{2}\alpha \rho Ll_{t}^{2}\left(L\right)-\left(EI-\alpha EIL\right)l_{xxx}^{2}\left(L\right)\leq \eta_{0}{\left(l_{t}\left(L\right)-l_{xxx}\left(L\right)\right)}^{2}=\eta_{0}u_{\alpha }^{2}$$

where: $\eta_{0}>\max\left(\eta_{1},\frac{\eta_{1}\eta_{2}}{\eta_{2}-\eta_{1}}\right)$, $\eta_{1}=\frac{1}{2}\alpha \rho L$, $\eta_{2}=EI-\alpha EIL$.

According to Equations (31) and (32), we obtain Equation (33).

(33) $$\begin{array}{l} H_{t}\left(t\right)\leq -\left(\frac{3}{2}\alpha -2\alpha L^{2}-\frac{2\alpha L^{3}}{EI}\right)\int_{0}^{L}EIl_{xx}^{2}\left(x\right)dx-\frac{1}{2}\alpha \int_{0}^{L}\rho l_{t}^{2}\left(x\right)dx-\left(k_{d}-\alpha I_{m}-k_{d}\alpha \right)e_{t}^{2}\\ -\left(\alpha k_{p}-k_{d}\alpha -2\alpha EIL-2\alpha L^{2}\right)e^{2}-\left(k-\eta_{0}\right)u_{\alpha }^{2}\leq -\lambda_{0}\left(W_{1}+W_{2}\right)\leq -\lambda_{0}\frac{H\left(t\right)}{\alpha_{3}}=-\lambda H\left(t\right) \end{array}$$

The following constraints must be met to ensure the validity of Equation (33).

(34) $$\begin{array}{l} \varepsilon_{1}=\frac{3}{2}\alpha -2\alpha L^{2}-\frac{2\alpha L^{3}}{EI}>0,\varepsilon_{2}=\frac{1}{2}\alpha >0,\varepsilon_{3}=k_{d}-\alpha I_{m}-k_{d}\alpha >0,\\ \varepsilon_{4}=\alpha k_{p}-k_{d}\alpha -2\alpha EIL-2\alpha L^{2}>0,\varepsilon_{5}=k-\eta_{0}>0,\min\left(2\varepsilon_{1},2\varepsilon_{2},\frac{2\varepsilon_{3}}{I_{m}},\frac{2\varepsilon_{4}}{k_{p}},\frac{2\varepsilon_{5}}{m}\right)\geq \lambda_{0}>0,\lambda =\frac{\lambda_{0}}{\alpha_{3}} \end{array}$$

The solution for the inequality in Equation (33) is $H\left(t\right)\leq H\left(0\right)e^{-\lambda t}$. Therefore, the Lyapunov function H(t) is close to 0 if the condition H(0) is bounded.

According to Equation (21), $W_{1}+W_{2}\to 0$, therefore, e→0 and $e_{t}\to 0$, meaning $\theta \to \theta_{d}$ and $\theta_{t}\to 0$. Additionally, $l_{t}\left(x\right)\to 0$, $y_{t}\left(x\right)\to 0$. Moreover, according to Lemma 2,$y_{x}^{2}\left(x\right)\leq L\int_{0}^{L}y_{xx}^{2}\left(x\right)dx$, $y^{2}\left(x\right)\leq L\int_{0}^{L}y_{x}^{2}\left(x\right)dx$. Therefore, we can further deduce that y(x)→0. Moreover, the closed-loop system tends to be stable.

## 4. Numerical Simulation Analysis

Three simulation tests were performed to verify the performance and effect of the proposed control method. During simulation, the discrete time was $\Delta t=5\times 10^{-4}$ and discrete distance was Δx=0.01m. The system parameters and controller coefficients were set as shown in Table 1. The initial state of the system and the initial interference compensation were set to 0. The number of neurons in the input layer, hidden layer, and output layer of the RBF neural network was 5, 5, and 2, respectively. According to the actual range of the input layer x, the parameters of the Gaussian basis function of the hidden layer were set to: $c_{j}=\left[\begin{array}{ccccc} -2.5 & -1.5 & 0 & 1.5 & 2.5\\ -2 & -1 & 0 & 1 & 2\\ -1.5 & -0.75 & 0 & 0.75 & 1.5\\ -1 & -0.5 & 0 & 0.5 & 1\\ -0.5 & -0.25 & 0 & 0.25 & 0.5 \end{array}\right]$, $b_{j}=0.5$. In order to ensure reasonable results, the parameters and coefficients in the simulation scenarios 1–3 should be consistent.

Scenario 1: with the adaptive boundary control method [5].

Scenario 2: with the RBF neural network control method [8].

Scenario 3: with the proposed control method.

**Remark** **4** . *The design description of coefficients* $k_{p}$*,* $k_{d}$*, and* *in control law is shown in Appendix A.*

**Remark** **5.**  *An appropriately large control gain*$k_{p}$ *can ensure the good tracking performance of the system, but if too large, the system will become invalid. Therefore, in practical operation, the system performance and the actuator saturation should be considered simultaneously when we design the coefficient.*

**Figure 3:**
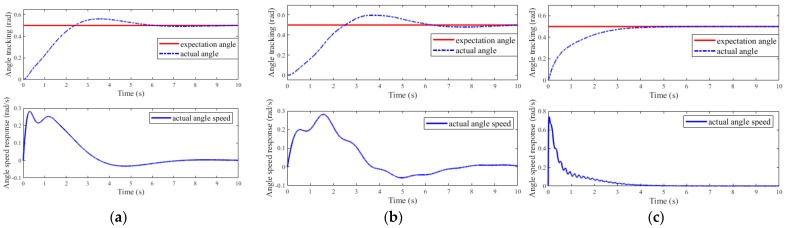
The angle tracking and response results of different methods: (**a**) adaptive boundary method; (**b**) RBF neural network method; (**c**) the proposed method.

**Figure 4:**
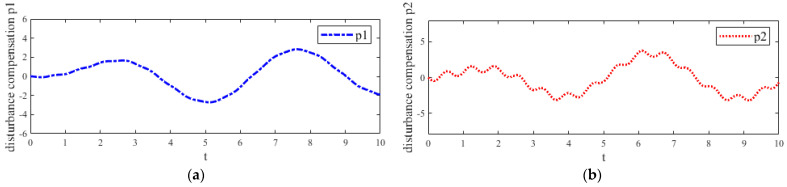
RBF neural network compensation results: (**a**) compensation results of *p_*1*_*; (**b**) compensation results of *p_*2*_*.

**Figure 5:**
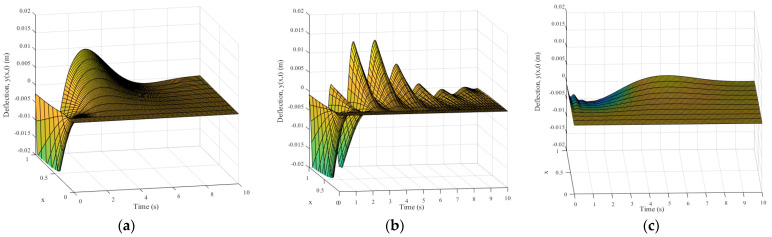
The deformation results of different methods: (**a**) adaptive boundary method; (**b**) RBF neural network method; (**c**) the proposed method.

**Figure 6:**
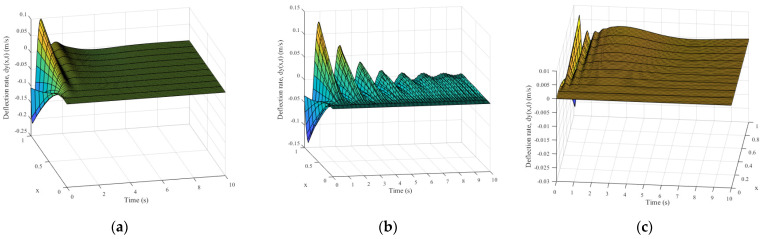
The deformation rate results of different methods: (**a**) adaptive boundary method; (**b**) RBF neural network method; (**c**) the proposed method.

**Figure 7:**
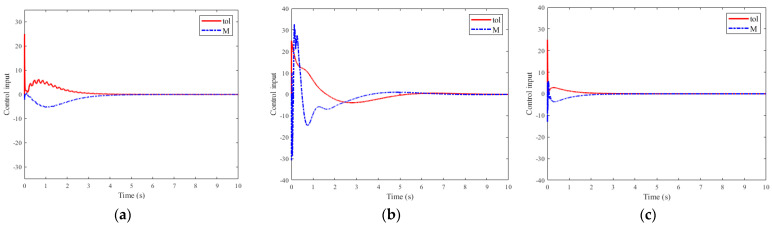
The control input *τ* and *M* results of different methods: (**a**) adaptive boundary method; (**b**) RBF neural network method; (**c**) the proposed method.

The simulation results of the micro-deformable manipulator with the proposed control method are shown in Figure 3c, Figure 4, and Figure 5c, Figure 6c and Figure 7c; results with the adaptive boundary control method are shown in Figure 3a and Figure 5a, Figure 6a and Figure 7a; Figure 3b and Figure 5b, Figure 6b and Figure 7b show the simulation results with the RBF neural network control method.

Figure 3 shows the angle tracking and angular velocity response results of micro-deformable manipulator with different methods. As shown in Figure 3a,b, the adaptive boundary control method and RBF neural network control method affect the control performance due to the large error and long response time. On the contrary, the proposed control method can accurately adjust the joint angle to the expected value within 6 s, as shown in Figure 3c. The response time comparisons of three methods are shown in Table 2. As shown in Table 2, the response speed is improved by at least 30% than that of the other two methods. Therefore, the compensation results of the RBF neural network are satisfactory, as shown in Figure 4.

Figure 5 and Figure 6 show the elastic deformation and deformation rate of manipulator successively. The statistical results of deformation rate are shown in Table 2. As shown in Figure 5a,b, the elastic deformation of the micro-deformable manipulator is remarkable, and it is not suppressed obviously. Conversely, in Figure 5c, the elastic deformation reaches the peak in 5 s and is eliminated obviously in 7 s. Furthermore, compared with Scenarios 1 and 2, the deformation rate of the manipulator is reduced by an order of magnitude with the proposed method (Table 2).

Figure 7 depicts the corresponding control input with different methods. Furthermore, the related statistical results are shown in Table 2. In Figure 7a,b, the control input of the adaptive boundary control method and RBF neural network control method is unacceptable because of sizeable fluctuation and overshoot. Conversely, in Figure 7c, the fluctuation and overshoot are nearly weakened within 2 s.

The three simulations confirmed that the proposed method performs excellently in trajectory tracking and vibration and deformation suppression of the micro-deformable manipulator under the premise of considering the double boundary interference. Through the analysis of the above simulation results, the proposed method is superior to the adaptive boundary control method and RBF neural network control method. Compared with the other two methods, the response time of the proposed method is reduced by at least 30%. The deformation of the manipulator is restrained to a great extent.

## 5. Experimental Tests

**Figure 8:**
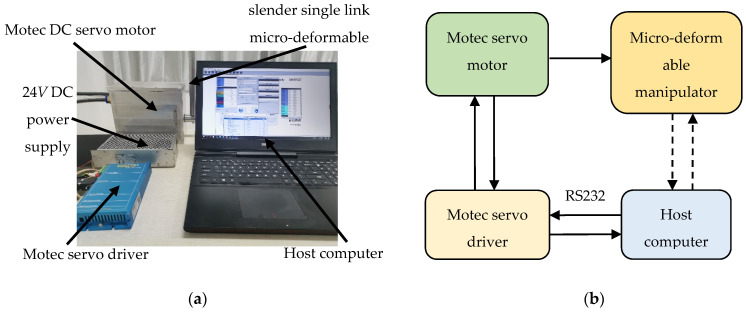
Experimental apparatus and schematic diagram: (**a**) experimental apparatus of the micro-deformable manipulator; (**b**) the schematic diagram of the experiment.

A simple slender single link micro-deformable manipulator (L = 1 m, R = 0.01 m which are consistent with the numerical analysis) was used for the experimental test in this paper. Air experiment was carried out in the laboratory. The experimental apparatus is shown in Figure 8a. In each experiment, the real-time position, current, speed, and other information of the micro-deformable manipulator were sent to the computer through the serial port, and then the computer, as the host computer, sent the order to the driver which drives the thruster to control the motion of the manipulator. The schematic diagram of the experiment is shown in Figure 8b. The thruster here was a MOTEC DC servo motor of DSEM-V241230E60LN type. It was driven by MOTEC DC servo driver of ARES-80-15-E-A0 type. The host computer here was a notebook computer with Intel Core i7-5500u 2.5 GHz CPU and 12 GB of RAM, running under Windows 10 operating system. The control software was developed by Visual Studio 2017. The manipulator system was powered by 24 V DC power supply.

**Figure 9:**
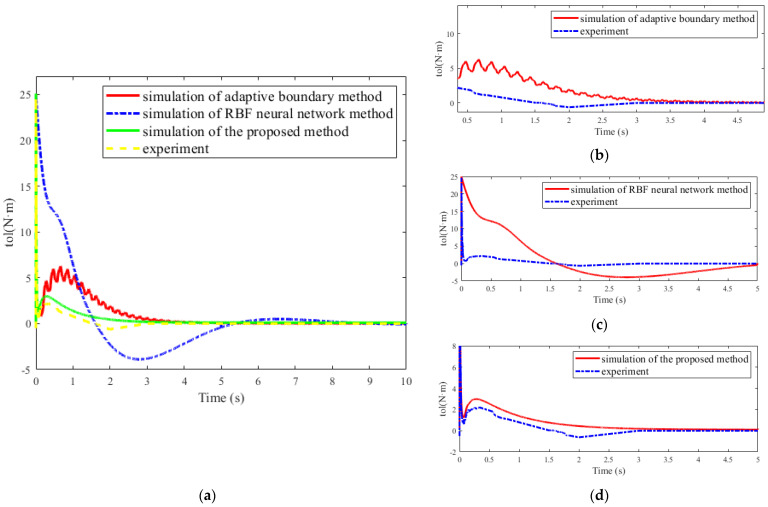
Comparison of τ between experiment and simulations: (**a**) comparison of overall results; (**b**) comparison of partial enlarged results of adaptive boundary method; (**c**) comparison of partial enlarged results of the RBF neural network method; (**d**) comparison of partial enlarged results of the proposed method.

In each air experiment, the parameters of the motor were set to pulse/2π rad in advance. The manipulator was controlled to rotate 1592 pulses per time, which was about 0.5 rad, and swing back and forth every time. In order to ensure the accuracy of data analysis, the angle set in the experiment was consistent with that in the simulation. The data of one cycle were collected for analysis (Figure 9).

Figure 9 depicts the change of the torque values of those collected from the experiment and simulated with different methods. The errors of experiment and three simulations are recorded in Table 3. In Figure 9b,c, the other two methods are not satisfactory due to their sizeable errors. As shown in Figure 9d, the variation of the numerical torque value of the proposed method is similar to that of the experimental torque value. The maximum error did not exceed 0.7 N·m as shown in Table 3, which verified the correctness of the numerical results of the proposed method.

## 6. Conclusions

This paper studied the PDE dynamic model; based on this model, the distributed boundary PD controller with infinite-dimensional RBF neural network compensator was proposed. The RBF neural network was used to compensate for double boundary interference and uncertain interference. The boundary control can be used to obtain the angle tracking of the micro-deformable manipulator. At the same time, it effectively weakened the vibration generated by the micro-deformation manipulator during the movement. The response time of the proposed method was reduced by at least 30% compared with the adaptive boundary controller and the RBF neural network controller. The error between the simulated results and experimental results was no more than 0.7 N·m, which verified the accuracy of numerical results and the feasibility of the proposed method. Since the unknown interference distribution in the external space is random and unpredictable, it is essential to subdivide the spatially distributed interference further and study the corresponding compensation control method in the future.

## Figures and Tables

**Table 1:** Numerical simulation marks of micro-deformable manipulator system.

**Mark**	*EI*	*m*	*I_m_*	*L*	*ρ*	*k_p_*	*k_d_*	*k*	*λ*
**Value**	3	0.15	0.1	1.0	0.21	60	30	20	200
**Unit**	N·m^2^	kg	kg·m^2^	m	kg/m	/	/	/	/

**Table 2:** The statistical results of the simulation data of the proposed method and the comparison methods.

Comparisons	Adaptive Boundary Method	RBF Neural Network Method	The Proposed Method
Response time (s)	7	9	6
Maximum of deformation rate (m/s)	−0.2132	−0.1396	−0.0269
Mean value of M (N·m)	0.2964	0.7233	0.3333
Mean value of τ (N·m)	−0.3334	−0.8040	−0.3750

**Table 3:** The errors between experiment and three simulations.

Errors (N·m)	Adaptive Boundary Method	RBF Neural Network Method	The Proposed Method
Mean value	1.3190	6.6750	0.3008
Standard deviation	0.9203	8.5060	0.1604
Maximum	1.7620	20.930	0.6644

## Nomenclature

L	Length of micro-deformable manipulator, m
R	Radius of micro-deformable manipulator, m
ρ	Linear density of micro-deformable manipulator, kg/m
$I_{m}$	Center moment of inertia, kg·m^2^
EI	Bending stiffness of uniform beam, N·m^2^
τ	Motor control input torque at initial end point, N·m
M	Control input force of end load, N
m	Terminal load mass, kg
θ	Joint turning angle (excluding deformation),
*y*(*x*)	Elastic deformation of manipulator at point *x*, m
*l*(*x*)	Offset of micro-deformable manipulator in the inertial coordinate system, m
F	End force in the free-body diagram, N
T	Moment obtained by force analysis, N·m
$\sigma_{ave}$	Mean normal stress, N/m^2^
$\tau_{s}$	Shear stress, N/m^2^
A	Cross section area of micro-deformable manipulator, m^2^
$p_{1}$	Boundary interference of one side
$p_{2}$	Boundary interference of another side
${\hat{p}}_{1}$	The estimated value of $p_{1}$
${\hat{p}}_{2}$	The estimated value of $p_{2}$
$k_{p}$	The coefficient of control law
$k_{d}$	The coefficient of control law
k	The coefficient of control law
λ	Flexibility of micro-deformable manipulator

## Appendix A

Design explanation of $k_{p}$, $k_{d}$, and k.

1. According to the engineering trial method, the coefficient $k_{p}$ is taken as 60.
2. According to Equations (19)–(21), $0<\alpha <\frac{1}{\left(2L,2\rho L^{3}/EI,2\left(I_{m}+2\rho L^{2}\right)/k_{p},2\right)}$.
3. From $\eta_{1}=\frac{1}{2}\alpha \rho L$, $\eta_{2}=EI-\alpha EIL$, we obtain that $\eta_{0}>\max\left(\eta_{1},\frac{\eta_{1}\eta_{2}}{\eta_{2}-\eta_{1}}\right)$.
4. Take $k_{d}=30$ according to the condition: $\varepsilon_{3}=k_{d}-\alpha I_{m}-k_{d}\alpha >0$; take k=20 according to the condition: $\varepsilon_{5}=k-\eta_{0}>0$.
5. Verifying other conditions in Equation (34):
  $\varepsilon_{1}=\frac{3}{2}\alpha -2\alpha L^{2}-\frac{2\alpha L^{3}}{EI}>0$, $\varepsilon_{2}=\frac{1}{2}\alpha >0$, $\varepsilon_{4}=\alpha k_{p}-k_{d}\alpha -2\alpha EIL-2\alpha L^{2}>0$, $\varepsilon_{5}=k-\eta_{0}>0$.
6. Take $\alpha_{3}=1.5$ according to the condition: $2>\alpha_{3}>1$.
7. From $\min\left(2\varepsilon_{1},2\varepsilon_{2},\frac{2\varepsilon_{3}}{I_{m}},\frac{2\varepsilon_{4}}{k_{p}},\frac{2\varepsilon_{5}}{m}\right)\geq \lambda_{0}>0$, we obtain $\lambda =\frac{\lambda_{0}}{\alpha_{3}}$.
